# A mutation in the *CACNA1C* gene leads to early repolarization syndrome with incomplete penetrance: A Chinese family study

**DOI:** 10.1371/journal.pone.0177532

**Published:** 2017-05-11

**Authors:** Xin Liu, Yang Shen, Jinyan Xie, Huihui Bao, Qing Cao, Rong Wan, Xiaoming Xu, Hui Zhou, Lin Huang, Zhenyan Xu, Wengen Zhu, Jinzhu Hu, Xiaoshu Cheng, Kui Hong

**Affiliations:** 1 Department of Cardiovascular medicine, the Second Affiliated Hospital of Nanchang University, Nanchang, China; 2 Jiangxi Province Key Laboratory of Molecular Medicine, Nanchang, China; 3 Department of Forensic Medicine, Medical College of Nanchang University, Nanchang, China; Pennsylvania State University, UNITED STATES

## Abstract

**Background:**

Early repolarization syndrome (ERS) may be a near-Mendelian or an oligogenic disease; however, no direct evidence has been provided to support this theory.

**Methods and results:**

We described a large Chinese family with nocturnal sudden cardiac death induced by ERS in most of the young male adults. One missense mutation (p.Q1916R) was found in the major subunit of the L-type calcium channel gene *CACNA1C* by the direct sequencing of candidate genes. A concomitant gain-of-function variant in the sodium channel gene *SCN5A* (p.R1193Q) was found to rescue the phenotype of the female *CACNA1C*-Q1916R mutation carriers, which led to the incomplete penetrance. The functional studies, via the exogenous expression approach, revealed that the *CACNA1C-*Q1916R mutation led to a decreasing L-type calcium current and the protein expression defect. The decreased calcium current produced by the mutant channel was improved by isoproterenol but exacerbated by testosterone. The effects of *CACNA1C-*Q1916R mutation and testosterone on cellular electrophysiology were further confirmed by the human ventricular action potential simulation.

**Conclusions:**

Our results demonstrated that the loss-of-function *CACNA1C-*Q1916R mutation contributed to ERS-related sudden cardiac death, and the phenotypic incomplete penetrance was modified by the *SCN5A*-R1193Q variant and sex. These findings suggest that phenotypes of ERS are modified by multiple genetic factors, which supports the theory that ERS may be an oligogenic disease.

## Introduction

An early repolarization (ER) pattern on electrocardiography (ECG) is characterized by ST-segment elevation and a terminal QRS slur or notch with J-point elevation ≥1 mm in 2 or more contiguous inferior and/or lateral leads [1]. The ER has been reported to be associated with a risk of arrhythmic death [2–5]. Accordingly, this clinic entity of idiopathic ventricular fibrillation and sudden cardiac death (SCD) with ER pattern is called ER syndrome (ERS) [1, 6]. Although the underlying arrhythmic substrates of ERS are not completely understood, the gene mutations are now considered a major pathogenic factor for developing ERS.

In several recent studies, ERS has been described as a kind of heritable disease. However, only a few ERS-related genes have been identified, including the cardiac inward rectifier potassium channel gene *KCNJ8*; the cardiac L-type calcium channel (LTCC) subunit genes *CACNA1C* (encoding the α subunit of LTCC, Cav1.2α1C), *CACNB2b* and *CACNA2D1*; the sodium channel α-subunit gene *SCN5A*; and the ATP-sensitive potassium channels subunit gene *ABCC9* [7–12]. Previously, most of the ERS-causing gene mutations were detected in the sporadic cases. A typical ERS family with Mendelian inheritance is extremely rare, and the identified mutations hardly account for the clinical traits regarding male predominance and incomplete penetrance. Therefore, the current understanding of the genetic basis of ERS remains limited, and it has recently been proposed that ERS may be a near-Mendelian or oligogenic inheritance disease [13]. Here we studied a large ERS-related SCD family via the genetic analysis and the functional analysis. The results suggested that the incomplete penetrance of ERS could be modified by the interaction of digenic variations and sex.

## Methods

### Clinical evaluation

This study conformed to the principles outlined in the Declaration of Helsinki and was prospectively reviewed and approved by the Medical Research Ethics Committee of the Second Affiliated Hospital of Nanchang University. Written informed consent was obtained from all participants whose blood samples were collected for genetic tests. All studied subjects underwent clinical assessments and examinations, including 12-lead ECG, 24-hour ambulatory Holter monitoring, treadmill exercise test, ultrasound echocardiography, and laboratory blood tests were performed in the Second Affiliated Hospital of Nanchang University. The diagnosis of ERS was based on the ECG parameters measured by two independent cardiologists. The peak of a terminal QRS notch or onset of a terminal QRS slur served as the J-point [1].

### Variant analysis

Genomic DNA was extracted from peripheral lymphocytes using the RelaxGene Blood DNA System (TIANGEN Biotech^®^, Beijing, China). All exons and exon–intron boundaries of candidate genes, including *SCN5A*, *SCN1B*, *SCN3B*, *KCNJ8*, *ABCC9*, *KCNJ11*, *ABCC8*, *KCNQ1*, *KCNH2*, *KCND3*, *KCNE1*, *KCNE2*, *CACNA1C*, *CACNB2b* and *CACNA2D1* were amplified by polymerase chain reaction and analyzed by direct sequencing. The genomic DNA of 800 chromosomes from 400 healthy ethnically matched individuals were used as controls.

### Mutagenesis and cell transfection

The full-length human WT *CACNA1C* cDNA containing exon 8a (GenBank accession no. AJ224873) cloned in pcDNA3.1 was a kind gift from Dr. Ganxin Yan’s Laboratory (Jefferson Medical College, Thomas Jefferson University, Philadelphia, Pennsylvania). The mutagenesis was performed using a QuikChange II XL Site-Directed Mutagenesis Kit (Agilent Technologies, La Jolla, CA, USA) and the plasmids were transfected into Human Embryonic Kidney (HEK) 293T cells using Lipofectamine® 2000 reagent (Invitrogen™, Carlsbad, CA, USA). Detailed methods are given in the supporting information, S1 File.

### Cellular electrophysiology and drug intervention

The HEK293T cells were purchased from the Shanghai Cell Bank of the Type Culture Collection Committee of the Chinese Academy of Science (Shanghai, China) and authenticated using short tandem repeat profiling by the Cell Bank. L-type calcium current (*I*_Ca-L_) through wild-type (WT) or mutant LTCC in HEK293T cells was measured by the whole-cell patch clamp recording at room temperature with an EPC10 patch clamp amplifier (HEKA Elektronik, Lambrecht, Germany) as the protocol previously described [14]. Furthermore, testosterone (10 μM) and isoproterenol (10 μM) were used to determine the biological effects on the mutated *I*_Ca-L_, respectively.

### Membrane fraction isolation and Western blot

These experiments were performed as previously described [15]. Briefly, 24 hours after transfection, the protein samples (separate membrane and cytosolic samples) were extracted from cells using the temperature-induced phase separation method. A total of 100 μg lysates or subcellular fractions were separated using 6 to 10% sodium dodecyl sulfonate-polyacrylamide gel electrophoresis (SDS-PAGE), and then transferred to polyvinylidene fluoride (PVDF) membranes and probed with specific antibodies. The following antibodies were used: Ca_V_1.2α1C (Alomone Labs, Jerusalem, Israel), ATP1A2 (Proteintech) and glyceraldehyde-3-phosphate dehydrogenase (GAPDH) (Proteintech). Signals of blots were collected through a Bio-RAD ChemiDox XRS detection system and analyzed by Quantity One analysis software (Bio-Rad, Hercules, CA, USA).

### Immunofluorescence

The immunofluorescence staining images were obtained as previously described [15]. The transfected HEK293T cells was probed by Ca_V_1.2α1C antibody (Alomone Labs), and imaged with a Leica TCS SP5 confocal laser scanning microscopy (Leica Microsystems, Heidelberg, Germany). The detailed methods for immunofluorescence staining are given in the supplementary information S1 File.

### Cardiac myocyte modeling

The action potentials (AP) of endocardium and epicardium simulations were performed based on a human ventricular cell model developed by Tusscher and Panfilov [16]. The model was accessed from https://models.physiomeproject.org/workspace/tentusscher_panfilov_2006 (Jul 25, 2016). In this cell model, a stimulus frequency of 1 Hz, an amplitude of -52 pA/pF, and a duration of 1 millisecond (ms) was chosen to generate an AP of cardiac myocyte. The effects of mutant calcium channel were simulated by reducing the maximal conductance of *I*_Ca-L_ (G_Ca_) from 0.9 mS/μF to 0.63 mS/μF (by 30%) in a heterozygous pattern. The effects of testosterone (10 μM) treatment were modified by adjusting the electrophysiological experiment data. Consequently, testosterone reduced WT G_Ca_ from 0.9 mS/μF to 0.74 mS/μF (by 26%) and reduced mutant G_Ca_ from 0.63 mS/μF to 0.554 mS/μF (by 35%).

### Statistical analysis

Numerical data are presented as the means ± standard error of the mean (SEM) and were analyzed using the Statistical Package for the Social Sciences version 19.0 (SPSS19.0). Statistical differences were determined with t-tests for single comparison, and one-way analysis of variance ANOVA followed by the Student-Newman-Keuls test was used for multiple comparisons. P values <0.05 were considered significant.

## Results

### Clinical characteristics

**Fig 1:**
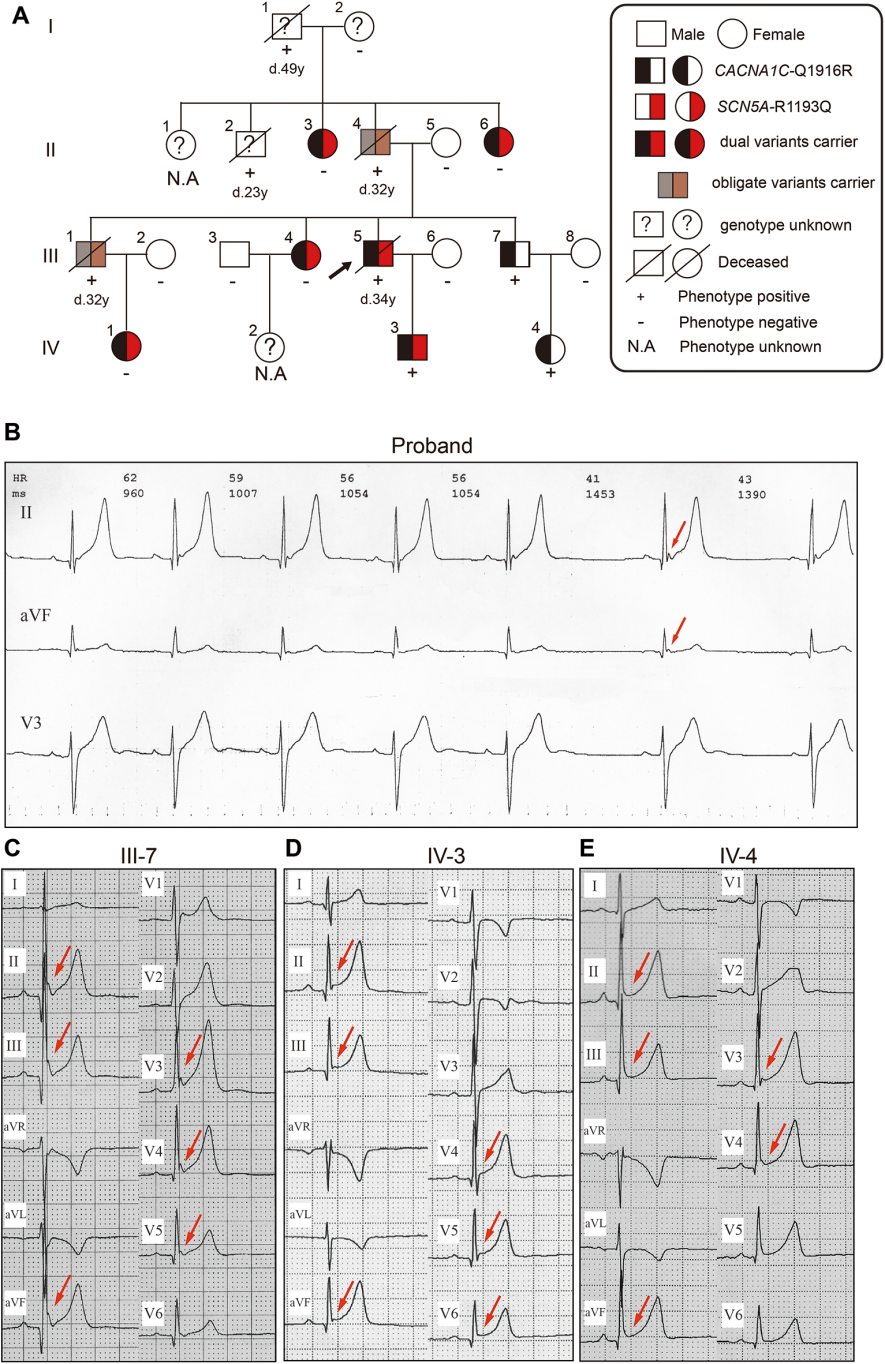
Pedigree and Holter (25 mm/s, 10 mm = 1 mV) results of the family. (A) Phenotypic and genotypic traits are represented by specific symbols in the pedigree. (B) Terminal QRS notch in leads II and aVF (arrows) without ST-segment elevation shown in the Holter of the proband (III: 5). (C) J-point elevation from 1 mm to 3 mm in leads II, III, aVF, and V3—V5 (arrows), which behaved as a terminal QRS notch without ST-segment elevation in III: 7. (d) Terminal QRS notch in leads III and aVF and concave ST-segment elevation (1 mm) in leads II, III, aVF, and V4-V6 (arrows) is presented for IV: 3. (E) Terminal QRS slur with ST-segment elevation in leads II, III, aVF, and V4, and a terminal QRS notch with ST-segment elevation in lead V3 with J-point elevation from 1.5 mm to 2 mm (arrows) is presented for IV: 4.

The pedigree of 4 generations of the Chinese family is presented (Fig 1A). Five seemingly healthy males (I-1, II-2, II-4, III-1 and III-5) died during sleep at a mean age of 34 ± 8.4 years old. The proband (III-5) suffered from nocturnal sudden death at age 34, and his autopsy report (provided by the Department of Forensic Medicine the Medical College of Nanchang University) revealed no pathogenic abnormality of the major organs. His previous medical records indicated that he had suffered from occasional dizziness and palpitations, but no chest pain or episodes of syncope had been observed. On his 24-hour ambulatory Holter monitoring, terminal QRS notches with J point ≥ 1.5 mm in leads II and aVF under the condition of bradycardia were documented (Fig 1B).

Of those affected adult males in this family, the proband’s 33-year-old younger brother (III-7), implanted with an implantable cardioverter defibrillator, was the only survivor. As shown in Fig 1C, the ER ECG pattern with terminal QRS notches in leads II, III, aVF, and V3-V5 (maximum J-point elevation 3 mm) was observed. Besides, the Holter tests of the other 2 surviving family members (IV-3, IV-4) manifested bradycardia-dependent J-point elevation with or without ST-segment elevation. In the Holter of the proband’s 7-year-old son (IV-3), concave ST-segment elevation (1 mm) in leads II, III, aVF, and V4-V6 and terminal QRS notches in leads III and aVF (J-point elevation: 1 mm) were observed (Fig 1D). The ST-segment elevation with terminal QRS slurs in leads II, III, aVF, and V4, and terminal QRS notches in lead V3 (maximum J-point elevation 1.5 mm) were also registered on the Holter of the 5-year-old daughter (IV-4) of III-7 (Fig 1E). The ECGs of other individuals (II-3, II-6, III-4 and IV-1) did not show any ER patterns (S1 Fig). The major ECG parameters of affected members are shown in Table 1.

**Table 1:** ECG characteristics of affected individuals.

Individual	sex	Age (yrs)	*CACNA1C*-Q1916R Carrier	*SCN5A*-R1193Q Carrier	HR (beats/min)	PR (ms)	QRS (ms)	J-point elevation (mm)	ST-segment elevation	QTc (ms)	Tp-ed (ms)	Tp-Te (ms)
II-4	M	32*	●	●	NA	NA	NA	NA	NA	NA	NA	NA
III-1	M	32*	●	●	NA	NA	NA	NA	NA	NA	NA	NA
III-5	M	34*	●	●	68	160	100	1.5	No	436	60	100
III-7	M	33	●		88	140	90	3	No	437	40	120
IV-3	M	7	●	●	80	160	80	1	Yes	413	20	80
IV-4	F	5	●		94	120	80	1.5	Yes	388	40	60

*Age of death

● positive variant carrier. M = Male; F = Female; NA = records not available; HR = heart rate; Tp-ed = Tp-e dispersion; Tp-Te = Tpeak-Tend.

### Genetic analysis

**Fig 2:**
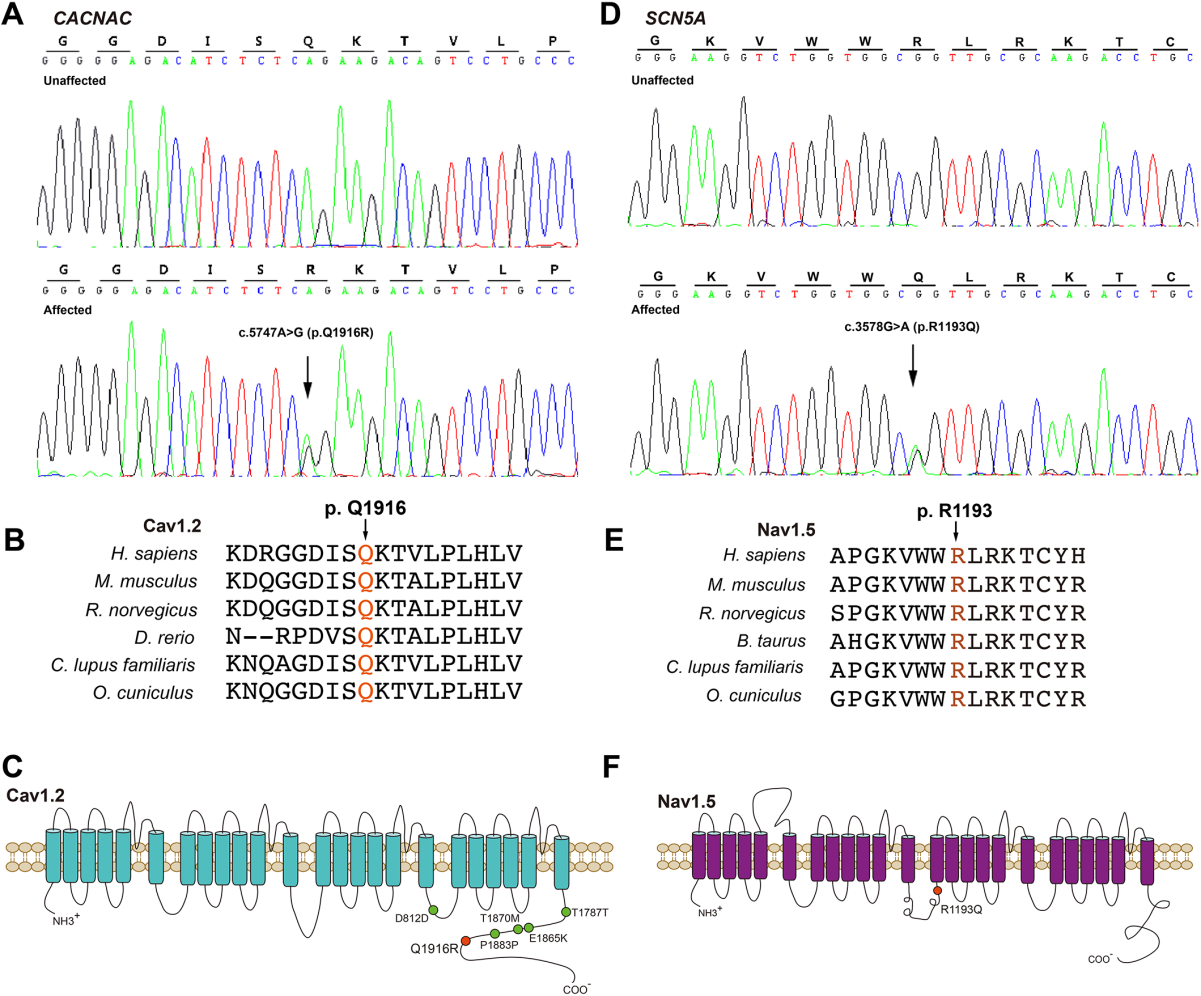
Gene variants of *CACNA1C* and *SCN5A* identified in the family. (A) Direct sequencing reveals a heterozygous mutation (c.5747A>G, p.Q1916R) in *CACNA1C*. (B) Amino acid sequencing alignments of *CANCA1C* indicate that Q1916 is highly conserved across mammals (red font). (C) Topology model of the α-subunit of LTCC. The localization of the mutation is indicated by a red dot, and polymorphisms are indicated by green dots. (D) A variant (c.3578G>A, p.R1193Q) in *SCN5A*. E. *SCN5A*-R1193Q is highly conserved among different species (red font). (F) Topology model of the sodium channel α-subunit. The localization of the polymorphism is indicated by a green dot.

A rare heterozygous mutation (c.A5747G, p.Q1916R) in *CACNA1C* located in the intracellular C terminus of Ca_V_1.2α1C with high evolutionary conservation was identified in the proband (Fig 2A–2C, Table 2). Five other common polymorphisms in *CACNA1C* with benign clinical significance were also detected (Table 2). The allele frequency of this variant was 0.000149 according to the Exome Aggregation Consortium (ExAC, http://exac.broadinstitute.org) [17] and 0.000799 (G) according to 1000 Genomes (http://www.internationalgenome.org/1000-genomes-browsers). Furthermore, this *CACNA1C*-Q1916R mutation was absent in 800 chromosomes from 400 healthy controls. A concomitant variant (p.R1193Q, rs41261344) with an allele frequency of 0.006215 (data form ExAC) in *SCN5A* was also found in the proband (Fig 2D, Table 2). This *SCN5A*-R1193Q variant, located in a highly conserved spot in the linker region between domains II and III (Fig 2E and 2F), has been reported to be “gain-of-function” and associated with long QT syndrome (LQTS) [18].

**Table 2:** The feature of the variants that identified.

Gene	Rs ID	Exon	Nucleotide change	Amino-acid change	Allele Frequency		Clinical significance
					1000 Genome	ExAC	(ClinVar)
*CACNA1C*	rs186867242	48	c.5747A>G	p.Q1916R	0.000799	0.000149	NA
	rs215976	17	c.2436C>T	p.D812D	0.14557	0.1621	Benign
	rs216008	30	c.3786C>T	p.F1262F	0.25919	0.2095	Benign
	rs1051375	44	c.5361G>A	p.T1787T	0.502636	0.6953	Benign
	rs201777030	47	c.5609C>T	p.T1870M	NA	0.003012	Benign
	rs56270948	47	c.5649G>A	p.P1883P	0.03594	0.02804	Benign
*SCN5A*	rs41261344	20	c.3578G>A	p.R1193Q	0.01238	0.006215	Pathogenic

ExAC = The Exome Aggregation Consortium; NA = Not available.

The result of sequencing screening revealed that all genotyped members with ancestry originating from I-1 and I-2 carried the *CACNA1C*-Q1916R mutation. Unfortunately, the genetic tests were not performed for the deceased II-4 and III-1, and therefore their genetic states were not defined. Given their offspring was mutation carriers, we might infer that II-4 and III-1 also harbored the *CACNA1C*-Q1916R mutation. Notably, not all *CACNA1C*-Q1916R carriers (II-3, II-6, III-4, III-5, III-7, IV-1, IV-3, IV-4 and obligate carriers II-4 and III-1) manifested the positive phenotypes (ER pattern in ECG or nocturnal SCD). This phenotypic incomplete penetrance might be modified by *SCN5A-*R1193Q variant and sex. As shown in Table 3, all male individuals carrying the *CACNA1C*-Q1916R mutation with (II-4, III-1, III-5 and IV-3) or without (III-7) concomitant *SCN5A-*R1193Q showed the ERS phenotypes. The female *CACNA1C*-Q1916R mutation carriers with *SCN5A*-R1193Q variant (II-3, II-6, III-4 and IV-1) were not affected, while the female member only carrying the *CACNA1C*-Q1916R mutation (IV-4) showed the ER ECG pattern. Other family members without the two variations had no ERS phenotypes.

**Table 3:** The correlation between genotype and phenotype.

Phenotype penetrance	*CACNA1C*-Q1916R +/-*SCN5A*-R1193Q +/-	*CACNA1C*-Q1916R +/-*SCN5A*-R1193Q -/-	*CACNA1C*-Q1916R -/-*SCN5A*-R1193Q -/-
Male	100% (4/4 cases)	100% (1/1 case)	0 (0/1 cases)
	II-4, III-1, III-5, IV-3	III-7	III-3
Female	0 (0/4 cases)	100% (1/1 case)	0 (0/4case)
	II-3, II-6, III-4, IV-1	IV-4	II-5, III-2, III-6, III-8

+/− heterozygous for variant; −/− wild type.

### Dysfunctional electrophysiology and drug intervention in mutated Ca_V_1.2α1C

**Fig 3:**
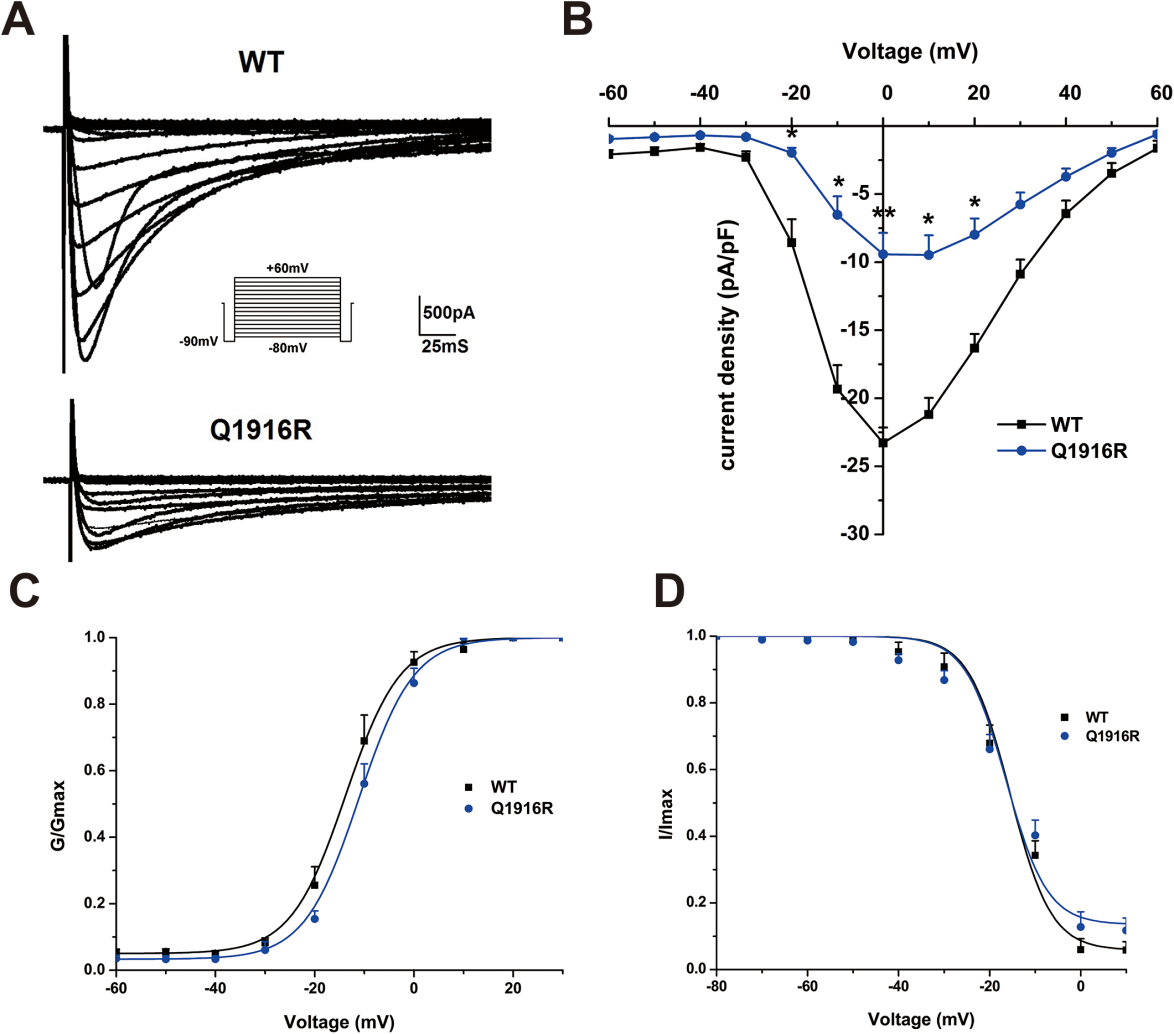
Macroscopic and kinetic characteristics of the mutant LTCC. (A) Representative whole-cell Ca^2+^ currents recorded from HEK293T cells transfected with WT or Q1916R mutation. Currents were elicited with the pulse protocol illustrated in the inset. (B) Current-voltage relationship for WT (n = 28) and Q1916R (n = 17) calcium channels. The peak Q1916R mutant *I*_Ca_ at 0 mV was decreased by 60%, when compared with the WT *I*_Ca_; values are the means ± SEMs (*P<0.05, **P<0.01). (C-D) The voltage-dependence of steady state activation (SSA) and steady state inactivation (SSI) did not significantly differ between the WT and Q1916R calcium channels.

To determine the molecular consequences of the *CACNA1*C-Q1916R mutation, we transfected *CACNA1*C with the other 2 subunits (*CACNB2b* and *CACNA2D1*) forming the LTCC into HEK293T cells and performed whole cell patch-clamp experiments. The Q1916R mutation reduced the amplitude of the current produced by LTCC when compared with the WT sequence (Fig 3A). The current density of the mutant LTCC over the voltage range of -10 mV to 20 mV was also reduced by 60% at 0 mV (-23.3±1.1 pA/pF for WT LTCC *versus* -9.4±1.6 pA/pF for mutant LTCC, n = 17–28, *P<*0*.*05*, **P<*0*.*01*; Fig 3B). However, the voltage-dependence of steady state activation (SSA) and steady state inactivation (SSI) were unchanged (Fig 3C and 3D). The results suggested that the *CACNA1*C-Q1916R mutation showed a “loss-of-function” in LTCC.

**Fig 4:**
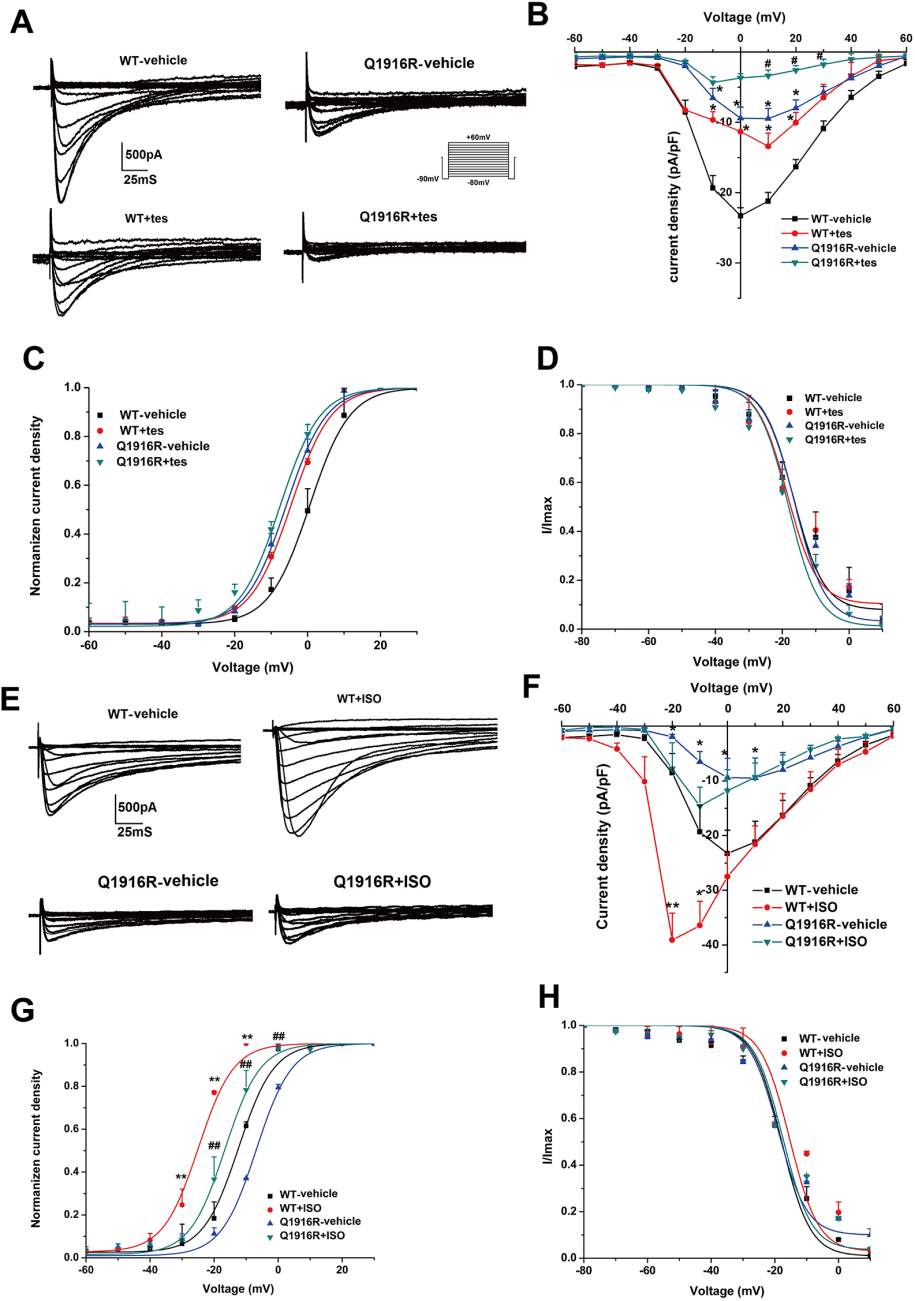
Effects of testosterone and isoproterenol on the WT and mutant LTCC. (A-B) The representative whole-cell Ca2+ current traces and average current-voltage relationship show a decrease in current density in both the WT (n = 6) and Q1916R (n = 8) LTCC after treatment with testosterone (*P<0.05, Q1916R or WT+tes *versus* WT-vehicle; #*P*<0.05, Q1916R+tes *versus* Q1916R-vehicle). (C-D) The voltage-dependence of SSA and SSI were not significantly altered among the 4 groups. (E-F) The representative whole-cell Ca2+ current traces and average current-voltage relationship show an increase in current density in the WT (n = 12) LTCC but not the Q1916R (n = 10) LTCC after treatment with isoproterenol (*P<0.05, **P<0.01, Q1916R or WT+ISO *versus* WT-vehicle). (G-H) The voltage-dependence of SSA displayed a left-shift in both the WT and Q1916R LTCC after treatment with isoproterenol, whereas no change was observed in SSI. SSA: steady state activation, SSI: steady state inactivation, tes: testosterone, ISO: isoproterenol.

To explore the underlying pathogenic role of sex and the possible therapy for the ERS with *CACNA1C*-Q1916R, HEK293T cells exogenously expressing the LTCC were treated with the principal male sex hormone, testosterone, or the non-selective β-adrenoceptor agonist, isoproterenol. As reported by Scragg JL *et al*., testosterone can directly interact with Cav1.2α1C protein and blockade the *I*_Ca-L_ in a concentration-dependent manner in the HEK293T cells [19]. The inhibitory effects of 10 μM testosterone were clearly independent of the activation voltage range, thus we chose 10 μM as a working concentration [19]. In the WT group, treatment with testosterone significantly reduced the current densities by 51.5% at 0 mV (-43.1±4.0 pA/pF for WT-vehicle group *versus* -20.9±0.04 pA/pF for the WT-testosterone group, n = 6, *P<*0*.*05*; Fig 4A and 4B). In the *CACNA1C*-Q1916R group, treatment with testosterone reduced the current densities by 69% at 30 mV (-11.4±1.7 pA/pF for the Q1916R-vehicle group *versus* -3.5±1.2 pA/pF for the Q1916R-testosterone group, n = 8, ^*#*^*P*<*0*.*05*; Fig 4A and 4B). Treatment with isoproterenol (10 μM) enhanced the current density of WT channels by 68.5% at -20 mV (-39.1±4.8 pA/pF for the WT-isoproterenol group *versus* -23.2±4.2 pA/pF for the WT-vehicle group, n = 12, *P<0.05) and mildly increased the current density of *CACNA1C*-Q1916R channels (-11.8±3.8 pA/pF for the Q1916R-isoproterenol group *versus* -9.5±3.5 pA/pF for the Q1916R-vehicle group, n = 10, *P*>0.05; Fig 4E and 4F). The voltage-dependent SSA displayed a left-shift in the Q1916R-isoproterenol group compared with that in the Q1916R-vehicle group, and the half-activation voltage (V_1/2_) shifted from -6.1 mV to -17.0 mV (*P*<*0*.*01*; Fig 4G). These results suggested an exacerbated role of testosterone but an improved effect of isoproterenol in the mutant LTCC.

### Decreased expression of mutated Ca_V_1.2α1C protein

**Fig 5:**
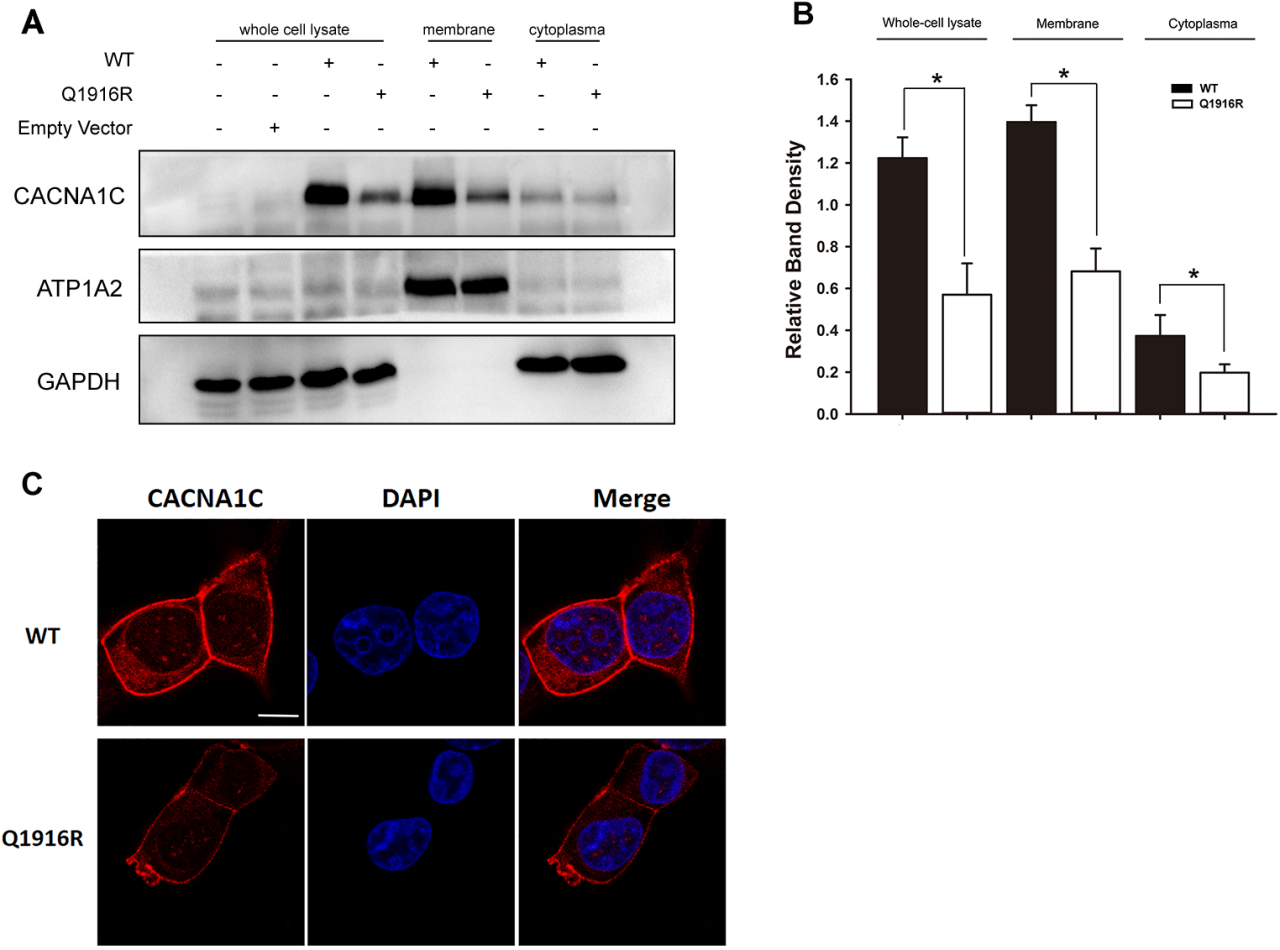
The expression and localization of the WT and *CACNA1C-*Q1916R channels in HEK293T cells. (A-B) Expression levels of WT and mutant of Ca_V_1.2α1C in the intact cell, membrane fraction and cytoplasmic fraction of the HEK293T cells. The left two lanes are blank controls with or without empty vector for CACNA1C. The expression of mutant CACNA1C was decreased in the intact cell, membrane and cytoplasmic lysates synchronously. The relative expression levels were normalized to GAPDH for both the intact cell and cytoplasmic lysate and were normalized to ATP1A2 for the membrane lysate. (n = 5, values are the means ± SEMs, *P<0.05, *CACNA1C*-Q1916R *versus* WT). (C) Confocal microscopy images of immunofluorescent staining in HEK293T cells. The red fluorescence indicated CACNA1C, and blue fluorescence indicated staining of the nucleus. The results showed that the intensity was reduced in the *CACNA1C*-Q1916R group.

To further study the mechanism of the decrease in Q1916R *I*_Ca-L_, protein expression and subcellular localization were detected in the exogenously expressing HEK293T cells. As shown in Fig 5A and 5B, the expression of mutant Ca_V_1.2α1C was reduced by 54% in the entire cellular lysate, 51% in the membrane fraction and 48% in the cytoplasm fraction, respectively, when compared with the expression of WT Ca_V_1.2α1C. In the similar manner, a decrease in the fluorescent intensity but normal localization was observed via immunofluorescence in HEK293T cells (Fig 5C).

### Human ventricular AP simulation

**Fig 6:**
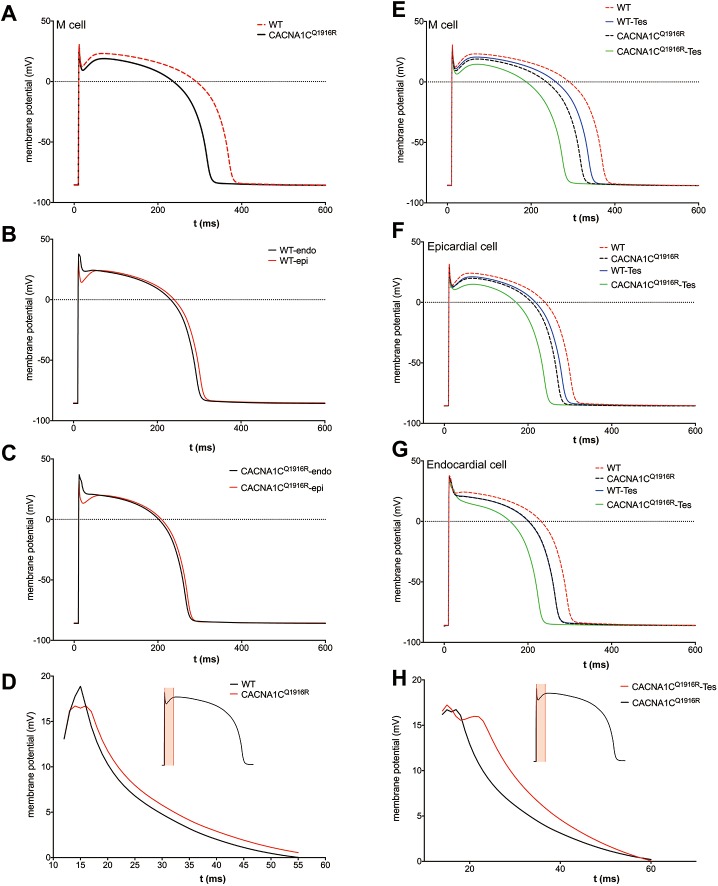
Single-cell ventricle AP modeling of three different cell types of myocardial layers. (A) Simulated AP of midmyocardial cell (M cell) in WT (red dashed line) and *CACNA1C*-Q1916R mutant condition (black solid line). (B-C) AP shape contrast between endocardial cell and epicardial cell in WT (B) or mutant condition (C). (D) Curves of the transmural voltage gradient between endocardial cell (endo) and epicardial cell (epi) in the WT condition (black) and the mutant condition (red) in the early repolarization stage (red shade region). (E-G) Effects of testosterone (Tes) on AP of M cell (E), epi (F) and endo (G). (H) Curves of transmural voltage gradient between endo and epi in the early repolarization stage (red shade region).

To evaluate the effects of *CACNA1C-*Q1916R mutation on the AP shape and the potential electrophysiological mechanisms of ER pattern formation, a human ventricular cell model was used to simulate APs with the *CACNA1C-*Q1916R mutation in the heterozygous status. In this model, *CACNA1C-*Q1916R was represented by a 30% reduction in the conductance of total *I*_*Ca-L*_. The APs of WT cardiac M (midmyocardial) cell and mutant M cell are shown in Fig 6A, in which a deeper notch of ER was observed in the mutant condition. Additionally, the APD90 (AP duration at the level of 90% of repolarization) was reduced by 14.29% in the mutant M cell. J waves are associated with the prominent voltage gradients between the endocardium and epicardium in the ER period. Accordingly, we compared the AP shape between the endocardial cell and the epicardial cell in WT or *CACNA1C-*Q1916R mutant condition, respectively (Fig 6B and 6C). Then, the transmural voltage gradient from the endocardial cell to the epicardial cell within the ER interval (from 12 ms to 55 ms of AP duration) was analyzed in the WT and the mutant conditions (Fig 6D). In the ER period, overall, the value of voltage gradient between endocardial cell and epicardial cell in the *CACNA1C-*Q1916R mutant condition was greater than that in the WT condition (16.1 mv for the *CACNA1C-*Q1916R mutation *versus* 14.44 mv for the WT at 18 ms).

We also modeled the effects of testosterone on the APs in these 3 cell types of cardiac tissue, according to the previous data of patch clamp experiment. The repolarization was earlier and the AP duration was shorter in the testosterone treated cell than that in non-treated cell, due to the more pronounced reduction of *I*_*Ca-L*_ (Fig 6E–6G). Testosterone enhanced the transmural voltage gradient (15.47 mv for testosterone treatment *versus* 9.93 mv for non-treatment at 23 ms) in the mutant cell (Fig 6H). These results highlighted that the ventricular transmural heterogeneity of electrical activity in the ER stage might be responsible for the generation of the ER pattern on ECG, and testosterone might aggravate the voltage gradient between different myocardium layers.

## Discussion

**Fig 7:**
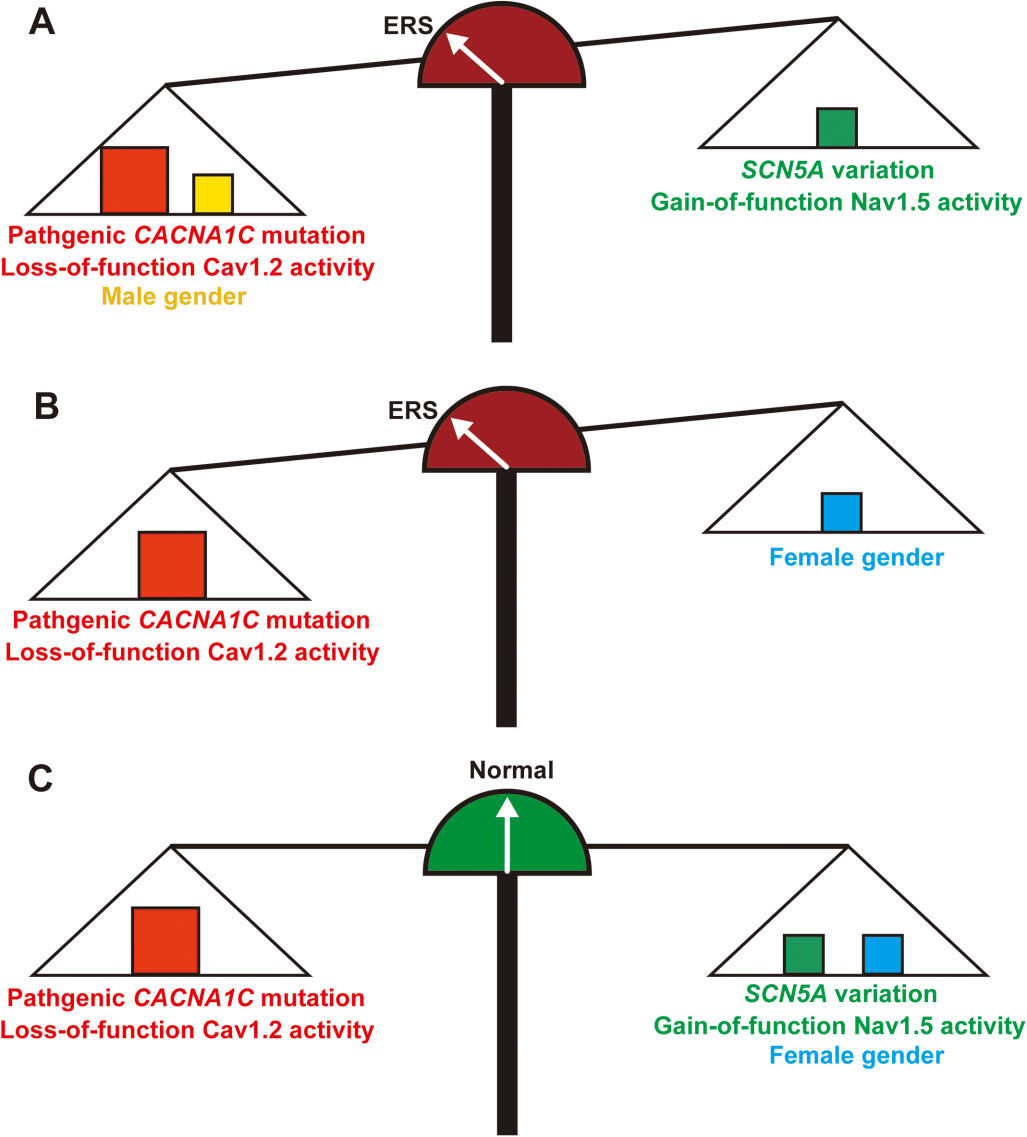
A graphical illustration of the balances represented by the relationships of three genetic contributors linked to the ERS phenotype in this study. (A) The disequilibrium represented the male patients with the *CACNA1C* mutation and *SCN5A* variation, the protective effect of the *SCN5A* variation was less than the pathogenic effects of *CACNA1C* mutation plus male sex, so the indicator turned pointed left toward the ERS phenotype. (B) The disequilibrium represented the female patients with the *CACNA1C* mutation. The pathogenic effect of the *CACNA1C* mutation was greater than the protective effect of female sex; therefore, the indicator pointed left toward the ERS phenotype. (C) The equilibrium state represented the female case with the *CACNA1C* mutation plus the *SCN5A* variation. The pathogenic effect of the *CACNA1C* mutation was neutralized by the *SCN5A* variation plus female sex, so the indicator consequently stopped in the middle pointing to the normal phenotype.

Here, we presented a rare large Chinese family with ER-associated SCD, in which the disease phenotypes were mainly caused by a *CACNA1C*-Q1916R mutation and modulated by the *SCN5A*-R1193Q variant and sex (Fig 7).

From the clinical perspective, ERS and Brugada syndrome (BrS) share some similar clinical traits, such as male predominance, mean age of first arrhythmic onsets, and circumstances of arrhythmic episodes. In genetics, loss-of-function mutations in *CACNA1C*, *CACNB2*, *CACNA2D*, *SCN5A*, and *SCN10A*, and gain-of-function mutations in *KCNJ8* have been shown to lead to both ERS and BrS. Hence, the two entities are referred to as J-wave syndrome by some investigators, but the concept is still controversial. The European Society of Cardiology (ESC) Guidelines for management of patients with ventricular arrhythmias and the prevention of SCD do not include the J-wave syndrome [20]. Furthermore, the differential response to sodium-channel blocker suggests that ERS and BrS are caused by the different basic pathophysiological mechanisms [21]. Indeed, the ST-segment elevation in the right precordial leads in BrS can be explained by abnormalities of both repolarization and depolarization in right ventricular outflow [22]. The affected family members were diagnosed based on the scientific statement referring to ERS, and no BrS was found during the familial clinical screening. Therefore, the mechanisms we studied here were specifically responsible for ERS rather than J-wave syndrome.

The ERS in this pedigree was inherited in an incomplete penetrance manner, with a penetrance of only 60%. The ERS caused by an *SCN5A* mutation G4297C also showed the inherited pattern of incomplete penetrance; however, the contributory factor for this phenomenon was not elucidated[10]. In another study of some small pedigrees with BrS caused by mutations in *CACNA1C*, male mutation carriers were more inclined to be symptomatic, which suggests that non-segregation of genotypes and phenotypes may be associated with sex [23]. BrS and ERS share a similar genetic complexity, and not all familial cases provide evidence for Mendelian inheritance [10, 24]. There are always multiple genetic and environmental factors that cause or modulate the phenotype; therefore, a more complex genetic inheritance known as the oligogenic model has recently been hypothesized to explain the particular clinical presentation of inherited cardiac disorders [25]. In this family, the evidence-based on the genetic and functional findings indicated that the loss-of-function mutation *CACNA1C*-Q1916R was the detrimental variation, and that the gain-of-function variant *SCN5A*-R1193Q modulated the phenotype. The candidate genes of ERS included *CACNA1C* and *SCN5A*, which suggests that the interaction of variations in these 2 genes may potentially modify the penetrance of ERS phenotypes.

As previously reported, *SCN5A*-R1193Q channels showed inactivation gating and generated a persistent, non-inactivating inward sodium current (*I*_Na_), which was associated with LQTS [18]. The QT interval on the ECG represents the depolarization and the repolarization phases of the cardiac AP. Decreases in repolarizing outward potassium currents or increases in depolarizing inward *I*_Na_ or *I*_Ca-L_ can result in the prolonged QT interval [26]. Type 3 LQTS is caused by the gain-of-function variation in the *SCN5A* (*e*.*g*., R1193Q), which evokes a sustained inward *I*_Na_ during the repolarization phase, leading to the deceleration of the repolarization and the prolongation of AP. In contrast, a decrease in *I*_Na_ or *I*_Ca-L_ or an increase in outward potassium currents may generate a prominent epicardial notch, which results in a greater transmural voltage gradient in the initiation of the repolarization phase. This voltage gradient registering as a J-point elevation on ECG may be clinically associated with lethal ventricular arrhythmias. The loss-of-function variation in *SCN5A* or *CACNA1C* (*e*.*g*., *CACNA1C*-Q1916R), which produces inadequate inward hybrid currents, is responsible for the pathopoiesis of ERS. Thus, from the mechanistic point of view, *I*_Na_ and *I*_Ca-L_ show a synergistic effect on the repolarization as two ingredients of the inward currents. In this study, we speculated that, during the repolarization phase, the inadequate inward current caused by the detrimental *CACNA1C*-Q1916R mutation might be partly compensated by the persistent inward tail *I*_Na_ produced by the *SCN5A*-R1193Q channel. That may be how *SCN5A*-R1193Q plays a protective role against the detrimental phenotype induced by the *CACNA1C*-Q1916R mutation. Unfortunately, additional experiment of an interaction between the rare mutation and the common variant was not feasible based on the *in vitro* experimental system that we used, the further *in vivo* study may be needed to confirm this assumption.

In the oligogenic model of ERS, sex is another high-impact contributor to the phenotypic differences. The male sex predominance has been observed in both the patients with ERS and healthy individuals with an ER pattern, and testosterone levels are significantly increased in men exhibiting an ER pattern than those without the ER pattern [27, 28]. The ER pattern was demonstrated to be the result of a transmural voltage gradient between the epicardium and endocardium, caused either by an increased outward ATP-sensitive potassium current (*I*_K-ATP_) or transient outward current (*I*_to_), or by a decreased *I*_Na_ or *I*_Ca-L_ [29, 30]. Testosterone has been reported to have a directly inhibitory effect on the *I*_Ca-L_ [19]. Our findings also suggest that male sex facilitates the formation of ER pattern under certain circumstances, and it is an independent risk factor of ERS in the family.

The *CACNA1C*-Q1916R variant was not considered to be "pathogenic", although it has been found in two small Japanese cohorts with inherited arrhythmias associated with sudden death [23, 31]. The ethnic formation of Eastern Asian subjects in the ExAC data set is not explicit, hence we cannot know the accurate incidence of *CACNA1C*-Q1916R in Chinese Han population based on ExAC. According to the data from the 1000 Genomes Project Phase 3, only 1 allele count is observed in Chinese Han population, and the allele frequency of *CACNA1C*-Q1916R in the Chinese population (including Chinese Dai in XiShuangbanna, Han Chinese in Beijing and Southern Han Chinese) is 0.0017 (1/601). Furthermore, in our study, the mutation was absent in 800 chromosomes from 400 healthy controls, which together indicates that *CACNA1C*-Q1916R might be considered a rare variant in the Chinese population, with a frequency lower than 0.001. We also found that the allele frequency of *CACNA1C*-Q1916R in the Japanese population was higher than that in other ethnic populations (Japanese: 3/208, Chinese: 1/601, African: 0, American: 0, European: 0, South Asian: 0) according to the 1000 Genomes data, which suggested that there might be a spatial diversity in the distribution of the *CACNA1C*-Q1916R mutation. In our study, the *CACNA1C*-Q1916R mutation was identified in a pedigree and was absent from the 800 control alleles. Additionally, *in vitro* experiments revealed that the Q1916R mutation impaired the electrophysiological function of the LTCC, which supported the known pathogenic mechanisms of ERS. Based on *Standards and guidelines for the interpretation of sequence variants*: *a joint consensus recommendation of the American College of Medical Genetics and Genomics and the Association for Molecular Pathology* [32], we consider *CACNA1C*-Q1916R a pathogenic variation.

The mechanism of ER-related ventricular arrhythmias involves the development of phase 2 re-entry derived from heterogeneous loss of the epicardial AP dome, which is accentuated by the elevated vagal tone due to the elevated *I*_to_ that is caused by the slow heart rate [29]. This explains why the arrhythmic events occur during sleep in ERS patients. Isoproterenol is widely used to suppress ERS-related VF, and the augmentation effect of *I*_Ca-L_ by isoproterenol may be one of its pharmacological actions [33, 34]. We observed that isoproterenol could enhance the activity of LTCC in the HEK293T cells, which may be associated with the evocation of cAMP/protein kinase A pathways by the activation of the endogenous β2 adrenoreceptors [35, 36].

In summary, we investigated an extremely rare large ERS family with a high incidence of nocturnal SCD, in which we found a pathogenic mutation in *CACNA1C* (p.Q1916R) with loss-of-function. The penetrance was also incomplete, which was modified by a gain-of-functional *SCN5A-*R1193Q variant and sex. The result strengthens the evidence that males are typically at higher risk of SCD, and the inheritance of ERS may be an oligogenic pattern rather than a monogenic model. The targeted mutation screening will effectively guide disease prevention and offspring fertility in the family. Isoproterenol, in theory, may be a putative pharmacologic option to improve the function of *CACNA1C*-Q1916R mutant channel. However, further translational studies are needed to explore the therapeutic feasibility to human. One limitation of our study is that the sodium channel blocker challenging test used for excluding BrS was refused by the family members. Another limitation is the lack of the induced pluripotent stem cell-derived myocytes from affected individuals and further *in vivo* study, which is preferable for interpreting the cumulative effects of complex genetic variants.

## Supporting information

S1 Fig Normal ECG of the family members. The ECG of II-3, II-6, III-4, IV-1 showed normal manifestation without ER pattern.
(TIF)

S1 File Details of methods. (DOCX)

S1 Data The original data relating to Figs 3–5. (XLSX)
