# Supplementary figures and images for: A mutation in the CACNA1C gene leads to early repolarization syndrome with incomplete penetrance: A Chinese family study
Source: PLoS One. 2017 May 11;12(5):e0177532. doi: 10.1371/journal.pone.0177532 (PMC5426766; doi:10.1371/journal.pone.0177532)

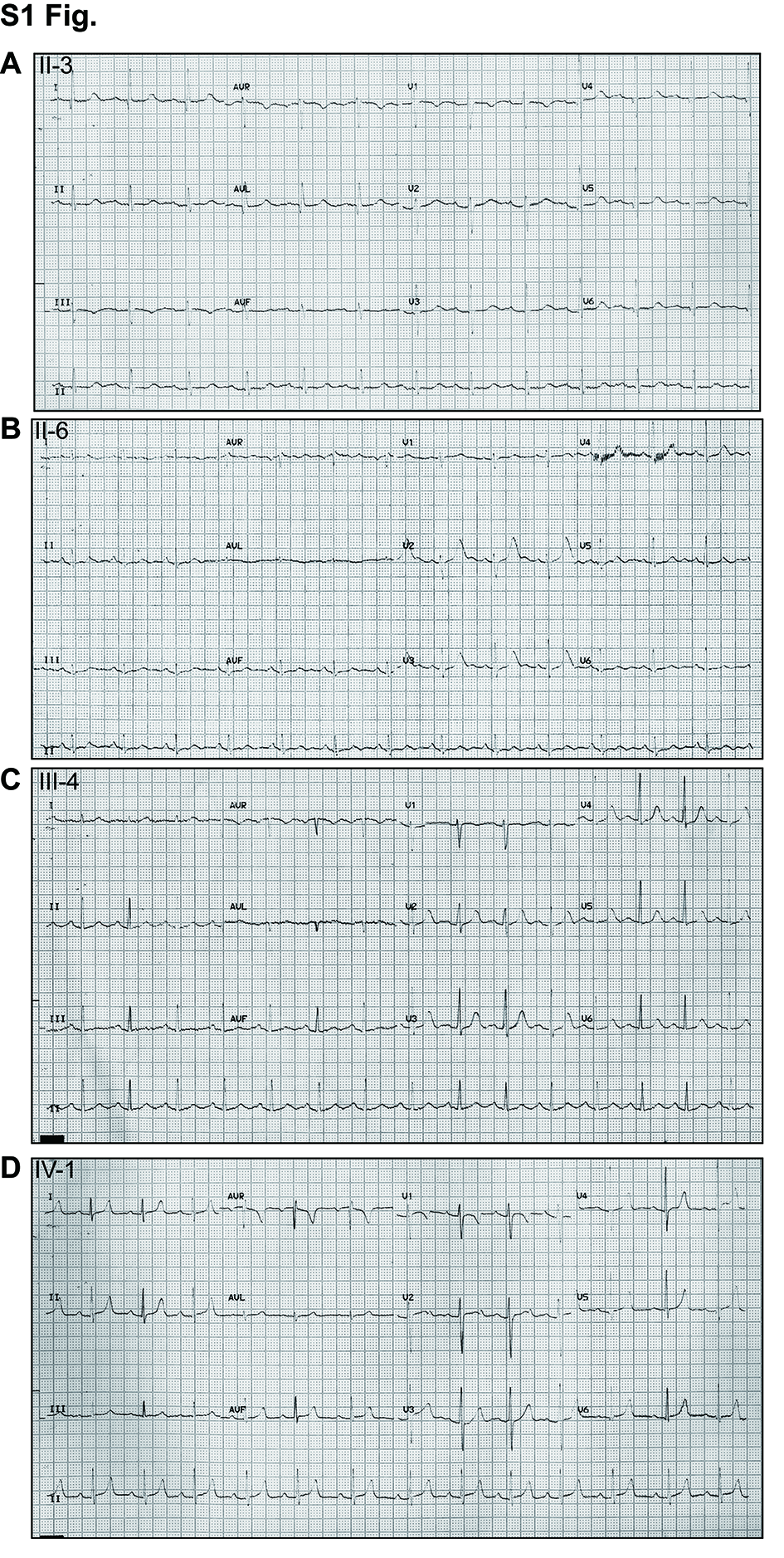

Supplement: S1 Fig — The ECG of II-3, II-6, III-4, IV-1 showed normal manifestation without ER pattern. (TIF) [file pone.0177532.s001.tif]
