# Supplementary material for: A mutation in the CACNA1C gene leads to early repolarization syndrome with incomplete penetrance: A Chinese family study
Source: PLoS One. 2017 May 11;12(5):e0177532. doi: 10.1371/journal.pone.0177532 (PMC5426766; doi:10.1371/journal.pone.0177532)
Supplement: S1 File — (DOCX) [file pone.0177532.s002.docx]

**SUPPORTING INFORMATION –S1**

**Materials and Methods**

**Mutagenesis and cell transfection**

The Q1916R mutation in CACNA1C was generated with mutagenic primers (forward: 5’-CATCTCTCGGAAGACAGTCCTGCCC-3’; reverse: 5’-CTGTCTTCCGAGAGATGTCTCCCCC-3’) and the QuikChange II XL Site-Directed Mutagenesis Kit (Agilent Technologies, La Jolla, CA, USA). Both WT and mutated clones were veriﬁed by sequencing to conﬁrm integrity and to ensure the presence of the target variant without other substitutions.

HEK 293T cells were transiently co-transfected with empty pcDNA3.1, CACNA1C-WT or CACNA1C-Q1916R and the auxiliary CACNB2b (encoding CaVβ2b) and CACNA2D1 (encoding CaVα2δ) at a 1:1:1 molar ratio by Lipofectamine® 2000 reagent (Invitrogen™, Carlsbad, CA, USA) according to the instructions. A plasmid that expresses green ﬂuorescent protein (pEGFP-N3) was added to positively visualize transfected cells for electrophysiology analysis. Cells were cultured for 48 to 72 hours before the expression or electrophysiological experiments.

**Immunofluorescence**

In brief, 48 hours after transfection, HEK 293T cells grown on cover slips were fixed with 4% paraformaldehyde and blocked in 5% bovine serum in PBS containing 0.1% Triton X-100. Then, the samples were incubated with a CaV1.2α1C antibody (Alomone Labs) in 1% BSA overnight at 4°C and incubated with an Alexa Fluor® 546-labeled secondary antibody in PBS for 1 hour at room temperature on the following day. After incubation, the samples were stained with 4',6-diamidino-2-phenylindole dihydrochloride for nuclear staining. The distribution of CaV1.2α1C was detected by Leica TCS SP5 confocal laser scanning microscopy (Leica Microsystems, Heidelberg, Germany).
